# How Can Flowers and Their Colors Promote Individuals’ Physiological and Psychological States during the COVID-19 Lockdown?

**DOI:** 10.3390/ijerph181910258

**Published:** 2021-09-29

**Authors:** Junfang Xie, Binyi Liu, Mohamed Elsadek

**Affiliations:** 1 Department of Landscape Architecture, College of Architecture and Urban Planning, Tongji University, Shanghai 200092, China; junfangxie@tongji.edu.cn (J.X.); 89098@tongji.edu.cn (B.L.); 2 Department of Horticulture, Faculty of Agriculture, Suez Canal University, Ismailia 41522, Egypt

**Keywords:** COVID-19 isolation, flower color, psychological effect, built environments, electroencephalography, restorative environments, stress, well-being

## Abstract

The global spread of COVID-19 has disrupted the normality of people’s daily lives, leading the population to social distancing and isolation. The closure of green areas also affected the well-being of the individual during the COVID-19 pandemic. Viewing flowers is expected to have similar positive effects to viewing natural scenery. Therefore, this study investigates how white, red, and yellow flower colors affect individuals’ psychological and physiological well-being. The experiment was conducted in an office-like setting with 50 participants. Participants looked at each flower color for 3 min. Electroencephalograms (EEGs), heart rate variability, and skin conductivity were measured to evaluate physiological responses along with both the semantic differential questionnaire (SD) and the Profile of Mood States (POMS) to assess psychological responses. EEGs showed that the mean values of alpha relative power in the prefrontal lobe were significantly higher when viewing yellow and red flowers vs. white flowers. Furthermore, heart rate variability revealed that viewing yellow and red flowers increased parasympathetic nerve activity significantly. After viewing the yellow and red flowers, the average results for each subscale of the POMS questionnaire improved. The vigor (V) subscale and overall mood status values were significantly improved. The results of the SD method revealed that viewing yellow and red flowers resulted in a significantly higher sense of relaxation, cheerfulness, and comfort than viewing white flowers.

## 1. Introduction

Green spaces can significantly impact people’s well-being [1,2,3], and many people prefer to visit them as frequently as possible for leisure, sports, relaxation, or socializing [1]. The COVID-19 pandemic, on the other hand, has dramatically changed the global population. Social isolation, one of the recommended practices to prevent disease spread, may contribute to developing various problems such as depression, stress, apathy, and loneliness [2]. In this extraordinary situation, people were forced to stay at home for several weeks, with no freedom of movement. Anxiety and depression (16–28%) and self-reported stress (8%) were among the most common symptoms in preliminary research on human psychological responses to the COVID-19 pandemic [3]. Furthermore, Bezerra et al. [4] found that having a house with an open space, like a terrace, or with a green space was beneficial during the isolation period.

Nature contact is linked with individuals’ health and well-being [5,6,7]. Several studies have found that humans exposed to nature have numerous psychological benefits (e.g., stress reduction, happiness, surgery recovery) as well as vascular disease reduction, increased physical exercise, and improved mental health [8,9,10,11]. A previous study explored the relationship between urban green space and well-being and found that people who live near green spaces have lower mental distress and higher well-being [12]. Increased exposure to or contact with natural environments (e.g., parks, forests, and gardens) has been linked to increased and better health and well-being [13,14]. Visual exposure to green spaces and plants has been linked to increased positive emotions such as relaxation, comfort, and cheerfulness, as well as decreased negative emotions like tension, depression, and stress [8]. These psychological advantages could be linked to the concept of biophilia (a preference for interacting in nature as a result of our evolutionary path) and could be based on restorative effects theories (i.e., recovery of psychological, social and physical abilities) [15]. Furthermore, there is evidence that living in spaces with few or no vegetation can lead to negative behaviors such as irritation or hostility [16]. These negative behaviors can be exacerbated by additional negative emotions such as fear, disappointment, and tension, which rise during the COVID-19 pandemic lockdown [17]. Incorporating natural materials, such as plants and flowers, can reintroduce nature into human living spaces and reconnect individuals to their natural surroundings.

People spend more than 80% of their time indoors, and their health is greatly affected by the indoor environment. On the other hand, much of the population lives in large urban areas on small properties with no garden space. Large building condominiums can have landscaped spaces; however, they have little or no use with social living restrictions. Therefore, many residents are looking for alternative ways to incorporate plants into their interior spaces. Indoor plants and flowers, in most cases, offer a way to get closer to nature. Furthermore, there has been an increase in interest in the health benefits of getting closer to flowers in modern stressful societies [18,19,20], but there have been few evidence-based studies on how people’s brain activity and heart rate variability change when they see flowers. 

As previously stated, there is clear evidence that nature and plants have a positive impact on an individual’s physiological (e.g., brain waves, heart rate variability, and skin conductance) and psychological (e.g., feelings and emotions) states associated with well-being [21,22,23,24,25]. Surprisingly, there has been little research into the effect of flower color on people’s emotional and physiological responses. Color has the power to change unpleasant feelings into positive feelings, to “level out” emotions, and to produce distinct moods [26]. According to some studies focusing on the behavior of florist shop customers, red flowers were the most preferred, and yellow flowers are the least preferred [27,28]. People, on the other hand, preferred red as their favorite color of tree canopy [29]. However, in another study, the red color was rated the lowest [30]. Several studies have found that red improves mental performance when compared to blue and green [31]. Others, on the other hand, have found the inverse of these findings [32]. As a result, understanding how different flower colors affect people’s physiological and psychological states is critical.

This study addressed the following research questions: Do flowers have a calming psychophysiological effect on humans? Can they influence people’s moods and emotions? Which flower color (white, red, or yellow) is most preferred over others? Despite an increase in the number of academics studying human–plant interaction, these evidence gaps persist. In order to respond to these queries, the current study aimed to determine the effect of seeing rose flowers with different colors in interior spaces on people’s physiological and psychological status. This research was carried out in a real office setting and included many measurements. The participants’ physiological responses, such as brain waves, heart rate variability, and skin conductance, were measured using an EEG machine and sensors, and their psychological responses were collected via questionnaires. The findings may contribute to a better understanding of how flowers and their colors affect humans in indoor spaces, as well as empirical evidence for a healthy indoor environment, which may aid interior landscape designers in selecting appropriate colors for various landscape decorations. The present study hypothesized that flowers and their colors can improve people’s well-being and reduce daily stress.

## 2. Materials and Methods

### 2.1. Participants

Fifty employees with an average age of 30.62 ± 1.20 years (mean ± SE), half of whom were male and half female, were voluntarily recruited to participate in the current study. Studies involving EEGs or other physiological measures with lower levels of variance in responses (vs. psychological reports on questionnaires) usually have a comparable number of participants to those recruited for this study [33,34]. Two steps were taken to recruit experimental volunteers: (1) An email was sent to the management of a company describing the study in detail, and (2) the authors of this research met the participants and invited them to take part in the experiment. All participants were Chinese and free of neurological disorders with normal visual acuity. The participants’ personal details and characteristics are mentioned in Table 1. The participants were instructed not to consume alcohol the day before the test to ensure physical and mental health during the experiment. During the study period, coffee and tobacco were also banned. All subjects gave their informed consent for inclusion before they participated in the study. The study was conducted in accordance with the Declaration of Helsinki, and the protocol was approved by the Ethics Committee of Tongji University (no. 2019 tjdx283). 

### 2.2. Visual Stimulation

**Figure 1:**
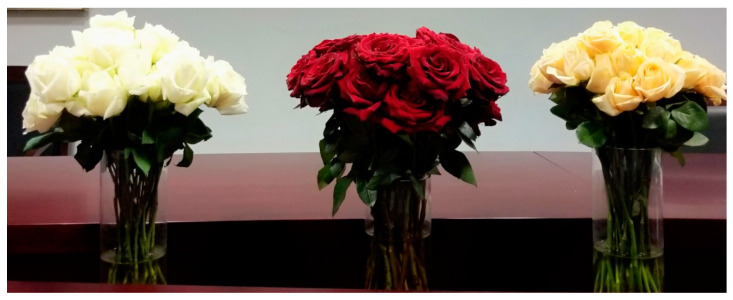
Visual stimuli, white, red, and yellow rose flowers.

Roses are the most popular cut flowers due to their beauty, variety, and long blooming season. Twenty-five fresh and unscented (sensory intensity graded as unscented) roses in white, red, and yellow colors were cut to 40 cm in length were used. The roses were arranged in a 15 cm diameter and 20 cm high cylindrical glass vase. The distance between the eyes of subjects and the flowers was 40–50 cm and adjusted by the subjects’ height. Figure 1 shows the flower colors used in the experiment.

### 2.3. Measurements 

#### 2.3.1. Physiological Measures

##### EEG Data Collection, Processing, and Analysis

**Figure 2:**
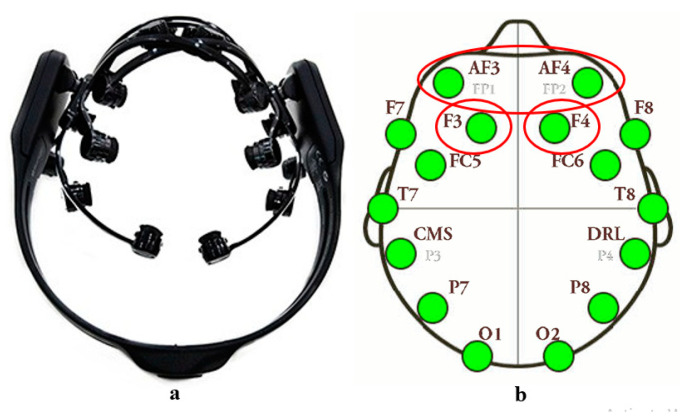
(**a**) EEG headset; (**b**) position of the electrodes.

An Emotiv EPOC wireless EEG headset was used to measure the EEG signal (see Figure 2). In previous landscape assessment studies, the reliability and validity of the EEG device used in the current study have been confirmed [35]. The Emotiv EPOC headset comprises 14 sensors placed in prefrontal (AF3, AF4, F3, F4, F7, F8), frontocentral (FC5, FC6), occipital (O1, O2, parietal) (P7, P8), and temporal locations (T7 and T8) in accordance with the International 10–20 Electrode System [36]. All felt pads had to be moistened entirely with a salt solution before use on top of the sensors. The recorded participants’ data were transmitted via Bluetooth to a laptop using a proprietary USB dongle with 2.4 GHz bandwidth. In order to ensure accurate measurement, the Emotiv Software Development Kit (SDK) offers package counting capabilities and a real-time contact sensor display. The EEG toolbox (<0.5 Hz or >50 Hz) was also filtered to removed electrical noise and other undesired signal functionality, such as DC offset, low-frequency skin potential artifacts, and high-frequency noise. 

Following collection, the data were put into MATLAB for further processing (version 7.12.0.635 R2011a). For each channel, the automatic independent component analysis technique ADJUST was employed to remove signal artifacts. ADJUST utilizes certain features of stereotypical spatial and temporal objects to identify and remove independent objects automatically [37]. Then, cleaned data were used to process the data. According to previous research [37], alpha waves (8–12 Hz) were evaluated in this study because they are closely related to human emotions and relaxation. Four prefrontal electrodes (AF3, AF4, F3, and F4) have been used to capture alpha relative wave power among the EEG electrodes. These electrodes were chosen for their role in controlling cognition and thinking [38]. The alpha relative power was calculated from 8 to 13 Hz by expressing absolute power in each frequency band as a percentage of absolute power over the two frequency bands [39] as shown in the equation below (1)

(1) $$\mathrm{RP}\left(f1,\;f2\right)=\frac{P\left(f1,\;f2\right)}{P\left(8,13\right)}\times 100$$

where P (.) stands for power, RP (.) for relative power, and f1 and f2 for low (LF) and high frequency (HF), respectively. The EEG headset and electrode channel position are shown in Figure 2.

##### Heart Rate Variability and Skin Conductance

In order to evaluate heart rate variability and skin conductivity, ErgoLAB synchronization platforms (Kingfar Inc. Beijing, China) have been utilized. The machine includes portable wireless sensors and a computer platform connected by a wireless receiver. The validity and reliability of this machine has been confirmed by researchers in related areas [40].

- Heart Rate Variability (HRV)

A portable wireless sensor was attached to each subject’s ear lobe using a PPG (Photoplethysmogram) to measure HRV at a sampling frequency of 64 Hz. HRV data were analyzed in frequency domains. High frequency (HF) power and low frequency (LF)/high frequency (HF) ratio were measured for the frequency domain. HF is primarily affected by parasympathetic activities, and the LF/HF ratio is sympathetic nerve activity [41]. Natural logarithmic transformed values were used in this study for standardizing participants’ HRV results.

b. Skin Conductance (SC)

In an earlier study, SC was an effective indicator of physiological stress and was usually used in stress recuperation studies [42]. SC is the dual-point conductivity on the skin surface that characterizes the electrodermal response of the skin and is a crucial indicator for the human body thermo-physiology research. The skin’s electrodermal status causes the secretion from sweat glands by two reusable electrodes connected with two fingers on one side. A wireless sensor with a range of 0 to 30 μs, 0.1 μs precision, and a sampling frequency of 32 Hz was used to measure SC directly. In two left-hand fingertips, two sensor electrodes were utilized.

#### 2.3.2. Psychological Measures 

Two psychological surveys, SD [43] and POMS [44], were used to assess the participants’ feelings and moods. The participants completed a POMS questionnaire before and after each task. The semantic differential questionnaire version in the Chinese language consisted of adjectives such as “comfortable–uncomfortable”, “natural–artificial”, and “relaxed–alert”. In the present study, six adjective items were selected, i.e., comfortable, relaxed, cheerful, beautiful, colorful and attractive, to examine participants’ psychological responses. Based on the level of emotion, each question had a 5-point Likert scale (−2, −1, 0, 1, and 2), with higher scores reflecting higher emotional status.

The POMS consist of 25 items, with six subscales: “tension/anxiety” (T-A), “depression” (D), “anger/hostility” (A-H), “fatigue” (F), “confusion” (C), and “vigor” (V). Total mood disturbance (TMD) was calculated according to the scores from each subscale using the following equation (2), lower TMD average indicating a higher emotional state.

*TMD* = ((*T*-*A*) + (*D*) + (*A*-*H*) + (*F*) + (*C*) − (*V*) (2)

### 2.4. Experimental Procedure 

**Figure 3:**
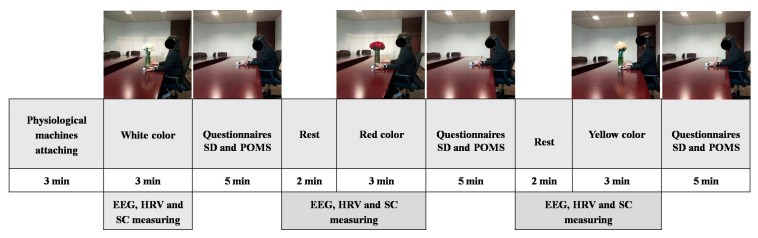
Experimental protocol.

The experiment began at 16:00, after the participants’ daily work had finished, to ensure that they needed to relax. Before the experiment began, all aspects of the experiment were explained to the participants, and they were informed that they could withdraw at any time. Forms of informed consent were signed by all participants. The participants were first asked to fill out a POMS scale. Following that, each participant was moved to the physiological measurements test room. During the experiment, the room temperature was kept at 25 °C and the relative humidity was kept at 60%. Before the participants entered the test room, the tested flowers were covered. Participants were asked to sit on a chair while the EEG electrodes and ErgoLAB measurement sensors were attached to their bodies. First, the participant was required to turn from opening to closing of the eye in four one-minute intervals to ensure accurate and stable electrode monitoring. The participants were then instructed to close their eyes and relax for two minutes to adjust their moods to the experimental environment. Participants were instructed to maintain a sitting posture, relax, and breathe evenly throughout the experiment. Then they were asked to open their eyes and focus on visual stimulation of either white, red, or yellow flowers for 3 min. Throughout the test procedure, continuous measurements of participants’ physiological reactions were taken. Then, each participant was asked to complete self-reported questionnaires, semantic differences (SD), and the Mood Profile (POMS). All participants were exposed to all conditions in a within-subject study design. The participants participating in an experiment were divided into three groups, and the order of the visual stimuli (white vs. red vs. yellow color) was counterbalanced among them. Figure 3 depicts the experimental protocol. Each participant spent 30–35 min on the experiment.

### 2.5. Statistical Analysis 

This study used a repeated-measures ANOVA and Bonferroni correction multiple comparisons to analyze the EEG, HRV, and SC data. A one-way ANOVA was used to analyze the SD data. The POMS data were analyzed using the Wilcoxon signed-rank test. The *p* value < 0.01 is considered significant. The data are shown as mean ± standard error.

## 3. Results

### 3.1. Electroencephalography 

**Figure 4:**
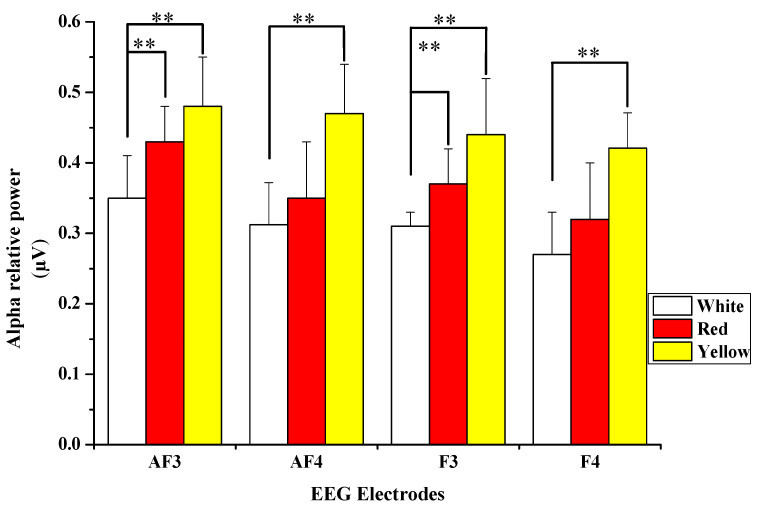
Alpha relative power variability in AF3, AF4, F3, and F4 electrodes while viewing white, red, and yellow flowers. ** *p*  <  0.01 is defined by Bonferroni correction.

Comparisons were made with alpha relative power averages of the four electrodes (AF3, AF4, F3, and F4) (see Figure 4). Significant differences in alpha relative power were observed when participants looked at the tested visual stimuli. Regarding AF3 channel, significant differences were observed between red: 0.43 ± 0.05 vs. white: 0.35 ± 0.06, *p* = 0.004 and between yellow: 0.48 ± 0.07 vs. white: 0.35 ± 0.06, *p* = 0.006. When participants viewed the flowers, the changes in the alpha relative power in the AF4 channel were significantly higher when compared with the white flower (yellow: 0.47 ± 0.07 vs. white: 0.31 ± 0.06, *p* = 0.009). In the F3 channel, however, there were significant differences between red: 0.37 ± 0.05 vs. white: 0.31 ± 0.02, *p* = 0.005 and yellow: 0.44 ± 0.08 vs. white: 0.31 ± 0.02, *p* = 0.000. When participants viewed the yellow flowers, the changes in the alpha relative power in the F4 channel were significantly higher compared to the white flower (yellow: 0.42 ± 0.05 vs. white: 0.27 ± 0.06, *p* = 0.009). This finding indicates that the visual stimulation of the rose colors yellow and red was responsible for the participants’ improved relaxation. Compared with viewing the white flower, when the participants saw both flower colors, the prefrontal lobe showed a significant emotional state response with higher alpha relative power.

### 3.2. Heart Rate Variability

#### 3.2.1. Parasympathetic Autonomic Nervous Activity

**Figure 5:**
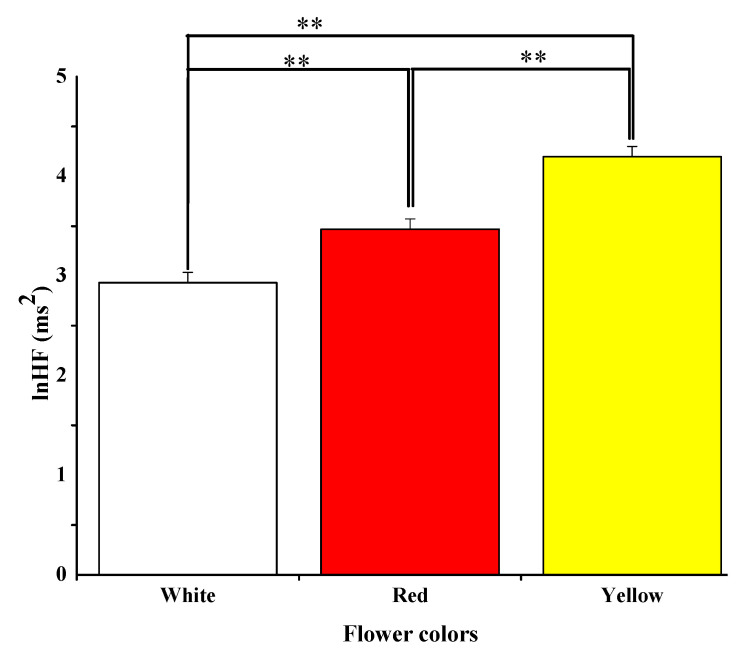
Average of the high-frequency component (HF) of heart rate when seeing white, red, and yellow flowers. ** *p* < 0.01 defined by Bonferroni correction.

Figure 5 represents the mean sum of ln(HF) values shown in the parasympathetic nerve activity when participants look at white, red, and yellow flowers. Viewing yellow and red flowers increased the values of ln(HF) significantly compared to viewing the white flower (red: 3.47  ±  0.11 ms^2^ vs. white: 2.93  ±  0.10 ms^2^, *p* = 0.007; yellow: 4.19  ±  0.10 ms^2^ vs. white: 2.93  ±  0.10 ms^2^, *p* = 0.009). Compared with the red flowers, viewing yellow flowers significantly increased the participants’ ln(HF) value (red: 3.47  ±  0.11 ms^2^ vs. yellow 4.19  ±  0.10 ms^2^ *p* = 0.001). The outcomes show that the relaxation state was higher when seeing yellow flowers than red and white flowers.

#### 3.2.2. Sympathetic Autonomic Nervous Activity

**Figure 6:**
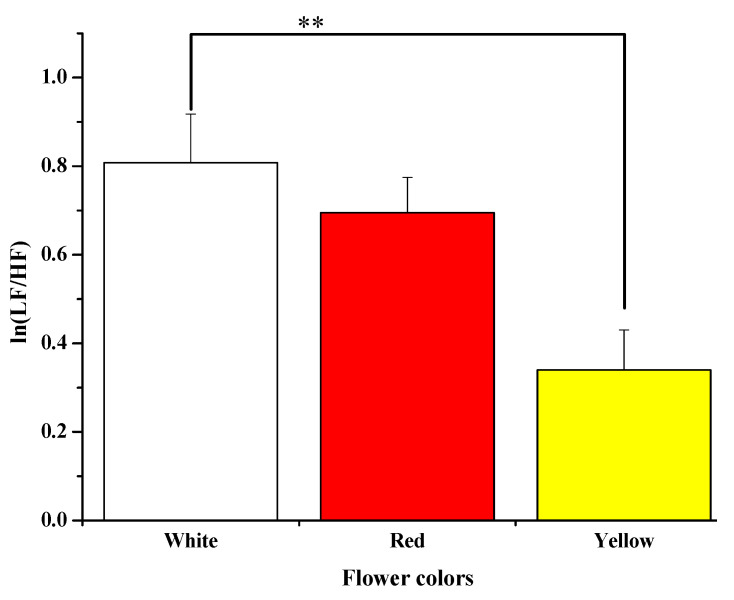
Average of ln(LF/HF) while seeing white, red, and yellow rose. ** *p* < 0.01 according to Bonferroni correction.

When participants saw the white flower, their mean ln(LF/HF) was significantly higher than when they saw the yellow flower (white: 0.80 ± 0.11 vs. yellow: 0.34 ± 0.09 *p* = 0.001). However, no significant difference was detected between the red and yellow flowers or between the red and white flowers (see Figure 6).

### 3.3. Skin Conductance

**Figure 7:**
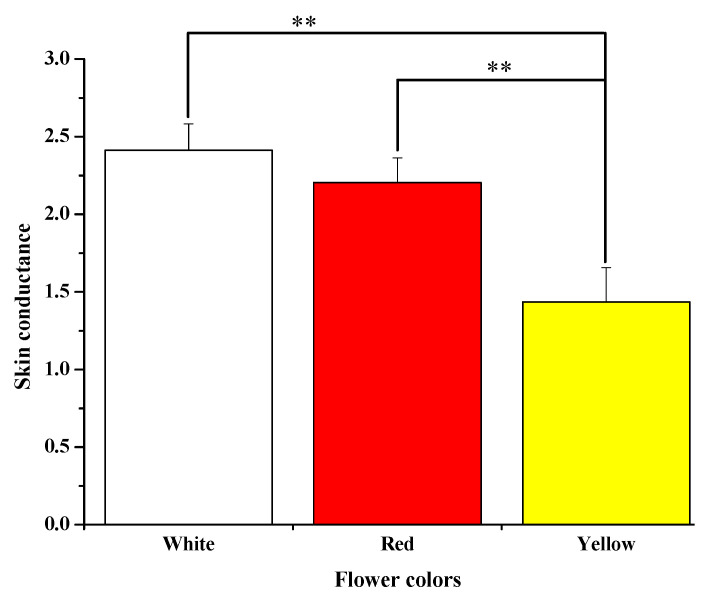
Average of skin conductance values recorded while seeing of rose colors (white, red, and yellow), ** *p* <0.01.

Figure 7 illustrates the average skin conductance of the participants over the 3 min experience period. When participants saw the white flower, the mean SC was significantly higher than when they viewed the yellow flower. The average skin conductance differed significantly between (white: 2.41  ±  0.17 vs. yellow: 1.43  ±  0.22, *p* = 0.000). However, a significant difference was found between viewing the red and viewing the yellow flowers (red: 2.20 ± 0.16 vs. yellow: 1.43 ± 0.22, *p* = 0.008). These findings show that yellow flowers positively affected participants’ physiological status due to a significant decrease in their SC compared to white and red flowers.

### 3.4. Psychological Responses

#### 3.4.1. Profile of Mood States

**Figure 8:**
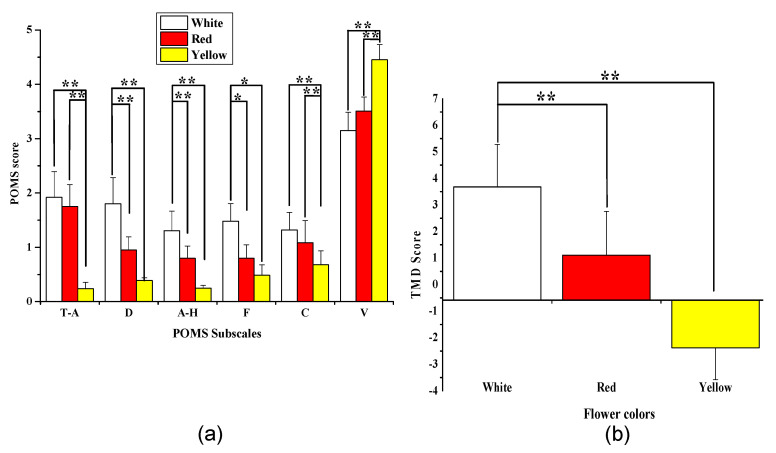
Subscale scores for the profile of mood states, tension/anxiety (T-A), depression/dejection (D), anger/hostility (A-H), fatigue (F), confusion (C), and vigor (V) after the visual stimulation with white, red, and yellow flowers (**a**); total mood disturbance (TMD) score **(b)**. * *p* < 0.05, ** *p* < 0.01 using the Wilcoxon signed-rank test.

Figure 8 depicts the participants’ mood states as reported in the POMS subscales. After viewing the red and yellow flowers, the negative subscales of POMS, “depression” (D), “anger/hostility” (A-H), “fatigue” (F) and were significantly reduced compared to the white flowers (*p* = 0.000), while “tension/anxiety” (T-A) and “confusion” (C) subscales were significantly decreased after viewing the yellow flowers compared with the white and red ones. In contrast, the “vigor” (V) subscale (*p* = 0.000) was significantly increased after visual stimulation with the yellow flowers. Furthermore, the score of TMD was decreased considerably after visual stimulation with the red (1.11 ± 1.64) and yellow (−2.38 ± 1.20) flowers compared with the white flowers (3.68 ± 1.60; *p* = 0.000). These results demonstrate that viewing yellow flowers and red flowers improves mood and evokes positive emotions more effectively than looking at white flowers.

#### 3.4.2. SD Questionnaire 

**Figure 9:**
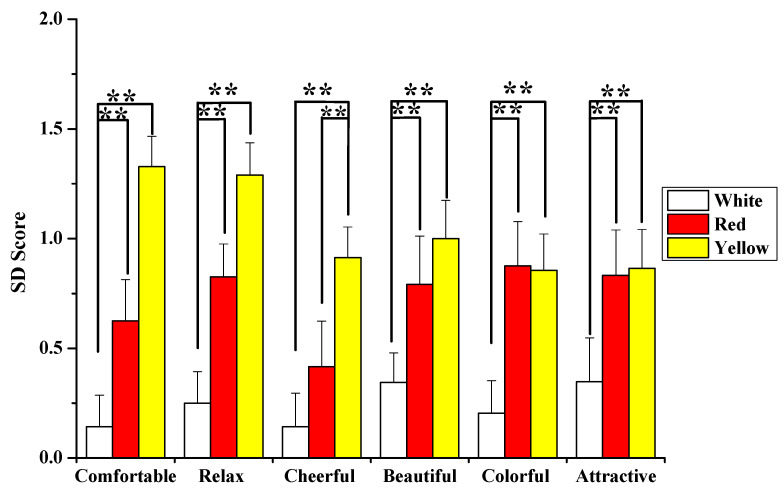
The participants’ emotions after viewing white, red, and yellow flowers, as measured by the semantic differential questionnaire. ** *p* < 0.01, using the Wilcoxon signed-rank test.

Figure 9 depicts the SD method’s results for measuring the participants’ emotions. Compared to the white flowers, participants felt more “comfortable” and “relaxed” after viewing red and yellow flowers. Additionally, they felt more “cheerful” after viewing the yellow flowers. Furthermore, the two rose colors, yellow and red, were rated as “beautiful” and “attractive”. Participants preferred these two colors over the white color (*p*  =  0.000). Given the findings, the presence of yellow and red flowers in the interior space is favorable. Meanwhile, as previously stated, participants’ psychological indices changed the most when they saw the yellow flowers. As a result, looking at yellow flowers can elicit more positive emotions than red flowers or white ones.

## 4. Discussion

Although the World Health Organization and the United Nations both agree that green spaces are necessary for healthy and livable environments [45], closing parks and green spaces limits this option for improving mental health during the COVID-19 pandemic. The natural environment has a significant influence on human physical and mental health, according to attention restoration theory [46] and stress reduction theory [47]. While plants are common in all public green spaces, interior plants are a less expensive and more easily accessible natural resource for city dwellers to access. Indoor plants can significantly increase contact time and intimacy with nature, improving physiological and psychological health [48]. The majority of existing research on the impact of plants on humans has focused on plant visual perception, but there is no evidence for associations between rose colors (white, red, and yellow) and individuals’ well-being during the COVID-19 pandemic. Here, the findings offer a 3 min vision recommendation for fresh flowers to improve human well-being. 

### 4.1. Effects on EEG

The current study found that viewing yellow and red flowers positively affects brain functioning more than viewing white flowers. Our findings revealed that alpha relative power was significantly higher when the participants viewed yellow and red flowers than when they viewed white flowers. This finding suggests that the visual stimulation of the yellow and red flowers is much greater than that of the white flowers and that it is more likely to cause relaxation or alertness. Alpha waves have been linked to reduced mental stress, increased sense of relaxation, and improved memory ability [49,50]. Furthermore, several studies have found that exposure to the natural environment increases alpha wave strength, linked to physiological relaxation and recovery effects [51,52,53]. This finding is consistent with Elsadek and Liu [20], who reported that looking at blue and purple flowers caused a significant increase in alpha relative waves in the prefrontal and occipital lobes. This would have made the participants more relaxed, alert, and focused on the environment around them. Notably, the current study’s increase in alpha relative waves when both flower colors are seen agrees with past relevant research. Previous research found that yellow flowers had a positive effect on the parietal and occipital lobes, indicating that subjects were calmer and happier [25]. Some studies, on the other hand, have discovered that flowers of different flowering periods or colors have a variety of influences on physiological responses [54,55]. Furthermore, Paraskevopoulou and colleagues conducted an experiment on the effect of seasonal color changes in plants and discovered that patients with psychotic disorders showed more positive facial expressions when viewing an image of a yellow-colored tree compared to a green-colored one [56]. Given the clear links between alpha wave power and relaxation [20,51] our findings suggest that viewing yellow flowers and red had a stronger restorative impact on participants than viewing white flowers. As a result, this study’s recommendations are to use yellow flowers or red flowers in places where a relaxing environment is required, such as offices, living rooms, and hospitals.

### 4.2. Effects on HRV and SC

Heart rate variability reflects the state of compassionate activation associated with stress and anxiety and, according to Malik’s research [57], parasympathetic activation reflects relaxation. Interestingly, it should be noted that viewing yellow and red flowers significantly increased the parasympathetic nerve activity, which induces a state of relaxation and significantly reduces the sympathetic nervous activity, thereby alleviating the stress state compared to viewing the white flowers. The results prove that seeing yellow and red flowers can improve relaxation and reduce stress. This study corroborates these previous conclusions [33,58]. The findings show that looking at red and yellow flowers for three minutes can improve people’s health and stress levels. According to previous research, looking at flowers has a calming effect. There was an increase in parasympathetic activity, decreased sympathetic activity, and a significant improvement in physiological recreation when the flowers were present [20,59]. A further indicator for determining the relaxation and stress of the participants is skin conductance. Several studies have revealed the link between the individual’s emotional state and skin conductance [60]. Previous literature shows that skin conductance rises with mental stress and emotional arousal [61,62]. When participants saw the yellow flowers, their skin conductance decreased significantly more than when they saw the red and white flowers, indicating that the yellow flowers were more effective for reducing stress. The findings are also in line with Kuper’s study, which found that people prefer yellow and red colors, which have been proved to improve psychophysiological states [63].

### 4.3. Effects on Psychological Responses

The current study’s POMS questionnaire results showed significant enhancement in average scores on each scale when seeing yellow and red flowers, with significant differences compared to white flowers. All negative POMS subscales significantly decreased after viewing yellow or red flowers compared with the white flowers. Meanwhile, the positive mood status index (vigor) improved considerably after viewing the yellow flowers. Low negative mood subscale scores have many positive results, which supports that the presence of flowers indoors can successfully reduce the stress and boost the vigor of people that stay inside for a long time. Furthermore, the mean TMD was significantly lower when seeing yellow flowers, indicating that yellow flowers induced a better emotional experience. The therapeutic effect of green spaces and plants is well documented [64,65]. Thus, the psychological benefits of viewing rose flowers may be therapeutic, as they are expected to alleviate stress, encourage positive mood states, and stimulate feelings of vigor, all consistent with previous findings [19,20]. As a result, the study’s findings showed the potential benefits of seeing flowers with different colors in our daily lives to promote positive emotions and reduce negative emotions.

In this study, the SD results revealed that the yellow flowers, followed by the red flowers, made the participants feel more “comfortable”, “calm”, and “cheerful”. SD results from a previous study revealed that participants who viewed the flowers felt more comfortable than those who did not [18,59]. Furthermore, these findings correspond to those found in previous studies [66,67].

### 4.4. Relationship between Physiological and Psychological Results

The current study attempted to introduce scientific evidence for the physiological and psychological benefits of seeing flowers in homes as one alternative solution for the population forced to stay at home for an extended period due to the COVID-19. The results of the alpha relative wave after seeing the yellow and red flowers were strongly correlated with the results of heart rate variability and skin conductance, indicating that yellow and red flowers positively influenced the participants’ physiological state. Furthermore, the positive subscales of POMS and SD increased, while the negative subscales decreased. These results revealed that both colors of roses improved physiological and psychological states. These physiological and psychological findings, taken together, are mutually supportive, implying that seeing yellow and red flowers appears to be more effective than looking at the white flowers in inducing calm and reducing stress symptoms. This study supports the growing body of scientific evidence showing the potential benefits of viewing different colors of flowers. The findings indicate that people who must stay indoors for an extended period, such as employees, city dwellers, and hospital patients, who cannot experience the benefits of the greenery by going outside, may also benefit from viewing and meditating on flowers. 

In other words, understanding how flowers can benefit people who spend most of their time indoors is critical. The participants tended to become calm during the visual stimulation with yellow and red flowers, according to the EEG, HRV, SC, and self-reported questionnaire results. Yellow roses were the most preferred, followed by red flowers; these results are consistent with previous research findings [68,69,70,71]. As a result, it is recommended that different fresh flowers of various colors be used in interior landscaping design to improve the physiological and psychological health of residents. This concept is especially important to promote in offices, hospital rooms, and other areas where there is a lack of green space.

### 4.5. Research Limitations

Despite these findings, there are some limitations to our experiment. First, because our study only included young participants, it is unknown whether the findings are applicable to other age groups and cultures. Performing this study with sample populations from a broader range of cultures would increase the generalizability of the study. Secondly, only three rose colors were examined; a broader range of flower colors and forms should be considered in future research.

## 5. Conclusions

The purpose of this study was to evaluate the effects of white, red, and yellow rose flowers on individuals’ physiological and psychological states using EEG, HRV, SC, POMS, and SD methods. The results suggests that 3 min of observation and meditation of yellow or red flowers might have strong positive immediate impacts on individuals’ well-being. The findings support the notion that seeing flowers can provide physiological and psychological advantages such as stress reduction and improved well-being. While flowers have a positive effect on people, the color of the flower impacts those positive effects as well. The yellow flowers, in particular, were most capable of improving the participants’ feelings of relaxation compared to the red and white flowers. 

## Figures and Tables

**Table 1:** Characteristics of participants (*n* = 50).

Variable	Average	Standard Error (SE)
Age (years)	30.62	1.20
Height (cm)	165.40	4.34
Body weight (kg)	58.92	5.64
Education	Higher education	
